# Correlation of the “quality of life” concept with related concepts

**DOI:** 10.1192/j.eurpsy.2026.12217

**Published:** 2026-07-31

**Authors:** A. M. Konovalova, S. D. Tomilovskiy

**Affiliations:** 1 Faculty of Psychology, Lomonosov Moscow State University; 2 Department of Pedagogy and Medical Psychology, Sechenov First Moscow State Medical University, Moscow, Russian Federation

## Abstract

**Introduction:**

The category of “quality of life” in modern scientific literature requires a clear distinction from related concepts such as “standard of living”, “welfare” and “well-being”.

**Objectives:**

To conduct a comparative analysis of the definition of “quality of life”, “standard of living”, “welfare” and “well-being”, identifying substantive overlaps and fundamental differences.

**Methods:**

The primary method employed is bibliographic analysis.

**Results:**

Quality of life is a comprehensive evaluation tool that characterizes various spheres of life. This concept is a broader indicator than material security, and encompasses both objective and subjective factors, such as health, life expectancy, environmental conditions, nutrition, household comfort, social environment, the fulfillment of cultural and spiritual needs, psychological comfort, etc. Quality of life as an indicator of individual socialization includes a person’s perception of their position in life in the context of the culture and value system in which they live, in accordance with goals, expectations, and norms.

The concept of “quality of life” in modern science requires a clear distinction from related concepts such as “standard of living”, “welfare” and “well-being”, since each of them has a specific meaning. The standard of living emphasizes material aspects, measured through income, housing and access to services, whereas quality of life encompasses both objective indicators (health, ecology, infrastructure), and subjective evaluation of satisfaction within the cultural and value context. Welfare, being an economy-centric category, focuses on the material security, while well-being reflects an individual’s internal psychological state, formed by external conditions and personal characteristics.

**Conclusions:**

Quality of life is a more comprehensive category, integrating economic, social, environmental and psychological aspects of human existence, which makes it a key focus of interdisciplinary research.

**Disclosure of Interest:**

None Declared

